# Skeletal Muscle Metastasis as an Initial Presentation of Follicular Thyroid Carcinoma: A Case Report and a Review of the Literature

**DOI:** 10.1155/2013/192573

**Published:** 2013-03-28

**Authors:** Mutahir A. Tunio, Mushabbab AlAsiri, Khalid Riaz, Wafa AlShakwer, Muhannad AlArifi

**Affiliations:** ^1^Radiation Oncology, Comprehensive Cancer Center, King Fahad Medical City, Riyadh 59046, Saudi Arabia; ^2^Histopathology, Comprehensive Cancer Center, King Fahad Medical City, Riyadh 59046, Saudi Arabia; ^3^King Saud bin AbdulAziz University for Health Sciences, Riyadh 11345, Saudi Arabia

## Abstract

*Introduction*. Follicular thyroid carcinoma (FTC) frequently metastasizes to the lungs and bones. However, metastasis to the skeletal muscles is an extremely rare manifestation of FTC. To date, only seven cases of FTC have been reported in the literature. Skeletal muscle metastases from FTC usually remain asymptomatic or manifest as swelling and are associated with dismal prognosis. *Case Presentation*. A 45-year-old Saudi woman presented with right buttock swelling since 8 months. Physical examination revealed right gluteal mass of size 13 × 10 cm and right thyroid lobe nodule. The rest of examination was unremarkable. Magnetic resonance imaging (MRI) showed 13 × 11.7 × 6.8 cm lobulated mass arising from the gluteus medius muscle, and tru-cut biopsy confirmed the metastatic papillary carcinoma of thyroid origin. The patient subsequently underwent palliative radiotherapy followed by total thyroidectomy and radioactive iodine ablation. At the time of publication, the patient was alive with partial response in gluteal mass. *Conclusion*. Skeletal muscles metastases are a rare manifestation of FTC, and searching for the primary focus in a patient with skeletal muscle metastasis, thyroid cancer should be considered as differential diagnosis.

## 1. Introduction

Thyroid cancer is the commonest endocrine malignancy, presenting with 23 500 and 19 000 new cases per year in the United States and the European Union, respectively [1, 2]. Differentiated thyroid carcinoma (DTC) is the most frequently diagnosed cancer among women in the Middle East, behind only breast cancer, and accounting for more than 10% of all cancers among women in Saudi Arabia [3]. 

Follicular thyroid cancer (FTC) is the second most common histologic type of DTC, and it commonly metastasizes to the lungs and bones. However, metastasis to the skeletal muscles is an extremely rare manifestation of FTC, only few related case reports have been reported in literature the [4]. Prognosis is generally dismal with reported median survival from 6–26 months.

Herein, we report a 45-year-old Saudi woman with a solitary metastasis to gluteus medius muscle as an initial manifestation of follicular variant of FTC. 

## 2. Case Presentation

A 45-year-old Saudi woman presented in our clinic with painful right buttock swelling and lethargy. She had noticed this swelling for 8 months, and it had been rapidly increasing in size over two months causing pain in sitting posture. Her previous medical history revealed type I diabetes mellitus since last 11 years and hypothyroidism since last 4 years; for those problems, she was taking thyroxin 50 micrograms daily and regular insulin. She had no history of trauma, smoking, and weight loss.

On physical examination, her vitals were stable. A fixed hard mass of size 13 × 10 cm was palpable in the right gluteal region. There was a moderate tenderness in the mass without any inflammatory surface, and there was no palpable inguinal lymphadenopathy. Neck examination revealed enlarged nontender right lobe of thyroid gland; however, no palpable cervical lymph nodes were noticed. Examination of chest, heart, nervous system, and abdomen was unremarkable. Differential diagnosis was soft tissue sarcoma or bone tumor. 

**Figure 1:**
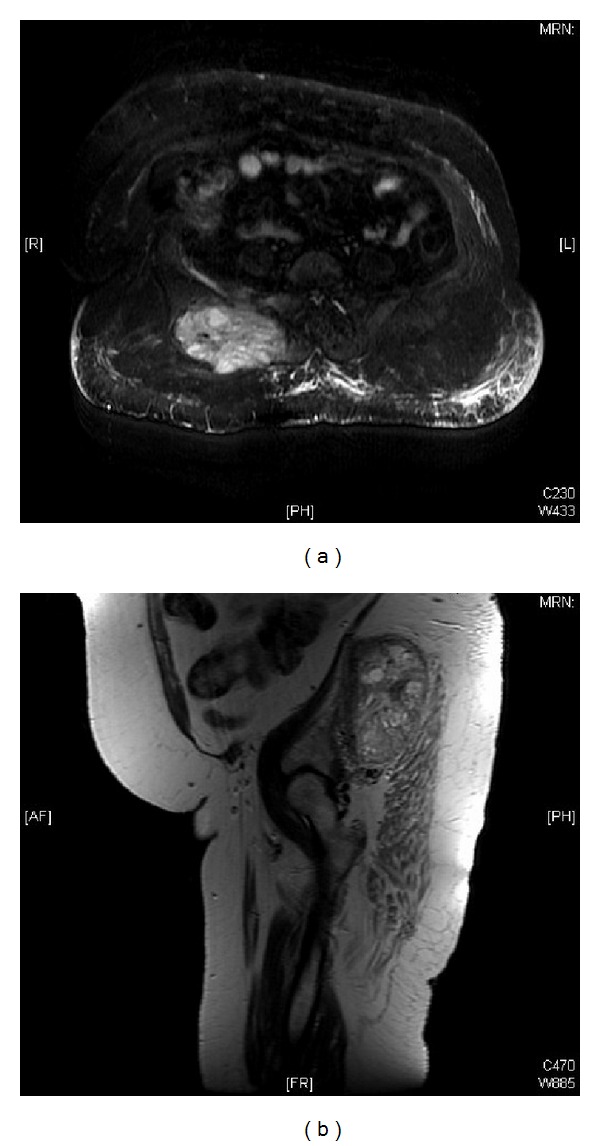
Magnetic resonance imaging (MRI) of the pelvis showing 13 × 11.7 × 6.8 cm lobulated heterogeneous mass in the right gluteus medius muscle also involving the right gluteus maximus, piriformis muscles extending to the right iliac bone and the right sacroiliac joint.

**Figure 2:**
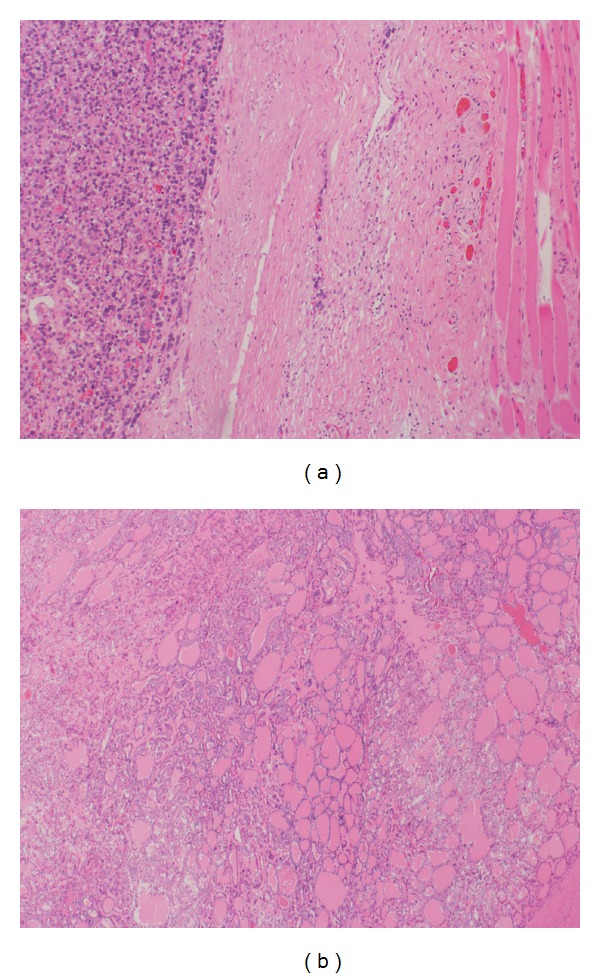
(a) Infiltrating clusters of papillary tumor cells in the skeletal muscle (H&E × 100) and (b) the follicular tumor cells (H&E × 200).

Hematology, serum electrolytes, and liver and renal function tests were within normal limits. Magnetic resonance imaging (MRI) of pelvis showed 13 × 11.7 × 6.8 cm lobulated heterogeneous mass in the right gluteus medius muscle also involving the right gluteus maximus, piriformis muscles extending to the right iliac bone and the right sacroiliac joint. The anteroposterior center of lesion was found within the right gluteus medius muscle; thus, the origin of the lesion was muscular rather than bony (Figure 1). Tru-cut biopsy of gluteal mass was taken. Histopathology revealed metastatic papillary tumor, and immunohistochemistry examination showed the positivity for Tg, and thyroid transcription factor-1 (TTF-1) made confirmed diagnosis of gluteal muscle metastasis consistent with FTC (Figure 2). Thyroid stimulating hormone (TSH) and thyroxin (T4) were found within normal limits; however, serum thyroglobulin (Tg) levels were raised, that is, 5632 ng/mL (normal: 5–25 ng/mL). Ultrasonography-guided fine-needle aspiration cytology (FNAC) of the right thyroid lobe nodule confirmed primary papillary carcinoma. Computed tomography (CT) on the chest and whole body iodine scintigraphy showed no other distant metastases.

**Figure 3:**
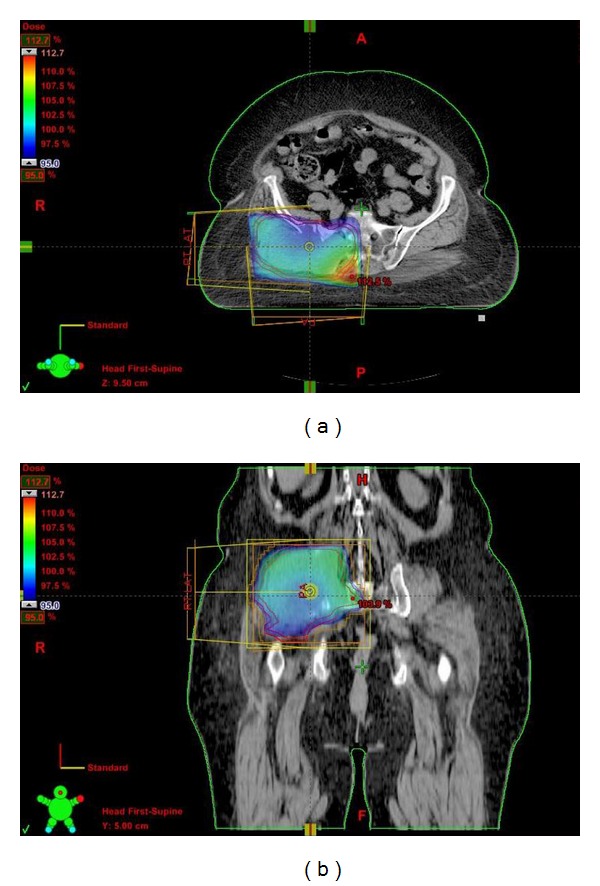
Palliative radiotherapy 30 Gy in 10 fractions to the right gluteal mass using posteroanterior (PA) and right lateral beams.

In multidisciplinary meeting, gluteal mass was labeled unresectable, and patient was referred to us for palliative radiation therapy. Patient received 30 Gy in 10 fractions to the right gluteal mass (Figure 3). After radiotherapy, her symptoms relieved and she underwent total thyroidectomy (pathological stage was T2N0; FTC), followed by radioactive iodine (RAI) ablation 200 mCi. At 9 months of followup period after the discovery of gluteal muscle metastasis, the patient was doing well with partial response in gluteal mass and complete response at primary origin.

## 3. Discussion

Skeletal muscle metastasis is a rare entity and differentiation between a primary soft tissue sarcoma and metastatic carcinoma is difficult without biopsy [10]. Most common malignancies metastasizing to skeletal muscles are lung, stomach, rectum, urinary bladder, and uterus. Skeletal muscle metastases present as painful masses of size 2–12 cm [11]. Skeletal muscle metastasis from FTC is an extremely rare manifestation; only 7 case reports have been published in the literature [4–9] (Table 1). 

Metastases of skeletal muscles may manifest as either isolated or with other metastatic sites and usually arise several years from initial diagnosis of FTC; however, in our patient the initial presentation was gluteal muscle metastasis. conventional iodine scintigraphy may remain fail to localize the foci of distant metastasis, because of the lack of anatomic landmarks. MRI, single photon emission computed tomography (SPECT), and positron emission tomography (PET) allow better location [9, 12]. 

Treatment is surgical resection followed by systemic therapy by RAI ablation or tyrosine kinase inhibitors (sorafenib or sunitinib) in noniodine avid metastasis [13]. Prognosis is variable with median survival from 12–24 months.

In conclusion, we have reported a case of FTC with gluteus medius muscle metastasis, which is extremely rare. MRI, SPECT, and PET are helpful to localize the site for metastatic deposits, and immunostaining, whenever possible, shall be incorporated to reach the final diagnosis and prompt treatment.

## Figures and Tables

**Table 1:** Cases of skeletal muscle metastasis secondary to thyroid carcinoma reported from 1990–2012.

Author	Age	Site	Variant	Treatment	Survival after diagnosis of skeletal metastasis
Bae et al. [4]	31 years female	Vastus medialis	Classical PTC	Surgical resection + RAI	24 months
Chaffanjon et al. [5]	53 years	Gluteus maximus	Classical PTC	Surgical resection + RAI	NA
Panoussopoulos et al. [6]	NA	Trapezoid	FTC	Surgical resection + RAI	NA
Bruglia et al. [7]	44 years male	Biceps femoris	Classical PTC	Surgical resection + RAI	24 months
Qiu et al. [8]	NA	Erector spinae	FTC	Surgical resection + RAI	NA
Zhao et al. [9]	NA	Rectus abdominis	Classical PTC	Surgical resection + RAI	NA
Present case	45 years female	Gluteus medius	FTC	EBRT + TT + RAI	Alive at 9 months

NA: not available, RAI: radioactive iodine ablation, EBRT: external beam radiation therapy, TT: total thyroidectomy, PTC: papillary thyroid carcinoma, and FTC: follicular thyroid carcinoma.
